# THE HEPATOPULMONARY SYNDROME

**DOI:** 10.1590/S0102-67202014000200012

**Published:** 2014

**Authors:** Lucas Souto NACIF, Wellington ANDRAUS, Rafael Soares PINHEIRO, Liliana DUCATTI, Luciana BP HADDAD, Luiz Carneiro D'ALBUQUERQUE

**Affiliations:** Disciplina de Transplante de Órgãos do Aparelho Digestivo, Laboratório de Investigações Médicas (LIM 37), Departamento de Gastroenterologia, Faculdade de Medicina, Universidade de São Paulo (Liver and Gastrointestinal Transplant Division, Department of Gastroenterology, University of São Paulo, School of Medicine), São Paulo, SP, Brazil

**Keywords:** Hepatopulmonary syndrome, Liver transplantation, Surgery

## Abstract

**Introduction:**

The hepatopulmonary syndrome has been acknowledged as an important vascular complication in lungs developing systemic hypoxemia in patients with cirrhosis and portal hypertension. Is formed by arterial oxygenation abnormalities induced from intrapulmonary vascular dilatations with liver disease. It is present in 4-32% of patients with cirrhosis. It increases mortality in the setting of cirrhosis and may influence the frequency and severity. Initially the hypoxemia responds to low-flow supplemental oxygen, but over time, the need for oxygen supplementation is necessary. The liver transplantation is the only effective therapeutic option for its resolution.

**Aim:**

To update clinical manifestation, diagnosis and treatment of this entity.

**Method:**

A literature review was performed on management of hepatopulmonary syndrome. The electronic search was held of the Medline-PubMed, in English crossing the headings "hepatopulmonary syndrome", "liver transplantation" and "surgery". The search was completed in September 2013.

**Results:**

Hepatopulmonary syndrome is classically defined by a widened alveolar-arterial oxygen gradient (AaPO2) on room air (>15 mmHg, or >20 mmHg in patients >64 years of age) with or without hypoxemia resulting from intrapulmonary vasodilatation in the presence of hepatic dysfunction or portal hypertension. Clinical manifestation, diagnosis, classification, treatments and outcomes are varied.

**Conclusion:**

The severity of hepatopulmonary syndrome is an important survival predictor and determine the improvement, the time and risks for liver transplantation. The liver transplantation still remains the only effective therapeutic.

## Abstract (pt)

**Introdução:**

A síndrome hepatopulmonar (SHP) tem sido reconhecida como importante complicação vascular nos pulmões desenvolvendo hipoxemia sistêmica em pacientes com cirrose e hipertensão portal. Ela é formada pela presença de anormalidade na oxigenação arterial induzida por dilatações vasculares intrapulmonares com a doença hepática e está presente em 4-32% dos pacientes com cirrose. Aumenta a mortalidade no cenário de cirrose podendo influenciar na frequência e gravidade. Inicialmente, a hipoxemia nos pacientes com esta síndrome responde à suplementação com baixo fluxo de oxigênio; mas, ao longo do tempo, há necessidade de maior suplementação de oxigênio. O transplante de fígado é a única opção terapêutica eficaz para a resolução.

**Objetivo:**

Atualizar conhecimentos sobre a síndrome hepatopulmonar, suas manifestações clínicas, diagnóstico e tratamento.

**Método:**

Foi realizada revisão da literatura com busca eletrônica realizada no Medline-PubMed em inglês cruzando-se os descritores “síndrome hepatopulmonar”, “transplante de fígado” e “cirurgia”. A pesquisa foi concluída em setembro de 2013.

**Resultados:**

Síndrome hepatopulmonar é classicamente definida por alteração no gradiente alvéolo-arterial (AaPO2) em ar ambiente (>15 mmHg, ou >20 mmHg em pacientes >64 anos de idade) com ou sem hipoxemia resultante da vasodilatação intrapulmonar na presença de disfunção hepática ou hipertensão portal. As manifestações clínicas, diagnóstico, classificação e tratamento são variados.

**Conclusão:**

A avaliação da gravidade da síndrome hepatopulmonar é importante meio de predição de sobrevivência e determinação do tempo e riscos para o transplante de fígado. O tratamento consiste em oxigênio suplementar e o transplante continua sendo a única terapêutica eficaz.

## INTRODUCTION

The hepatopulmonary syndrome (HPS) has been acknowledged as an important vascular complication in lungs due to systemic hypoxemia in patients with cirrhosis and portal hypertension. Is formed by a clinical triad of arterial oxygenation abnormalities induced by intrapulmonary vascular dilatations with liver disease. It is present in 4-32% of patients with cirrhosis^14,19^. It was also seen in both genders in middle-aged patients^17^. HPS pathogenesis is not well defined, but it is speculated that a combination of factors, such as an imbalance in the response of vascular endothelin receptors, pulmonary microvascular remodeling and genetic predisposition, leads to intrapulmonary vascular dilatation and bacterial translocation^1,10,12,14,17,19^.

HPS increases mortality in the setting of cirrhosis and may influence the frequency and severity. Initially the hypoxemia responds to low-flow supplemental oxygen, but over time, the need for oxygen supplementation is necessary. Currently, no pharmacological intervention can readily improve arterial oxygenation and alter the course of HPS. Thus, liver transplantation is the only effective therapeutic option for its resolution^13,14,17,19^.

The aim of this review is to update HPS on its clinical manifestation, diagnosis and treatment.

## METHODS

### Study identification and selection

The review was performed using electronic search on Medline-PubMed, in English. The search was performed through www.ncbi.nlm.nih.gov/pubmed and Mesh-term crossing the headings "hepatopulmonary syndrome", "liver transplantation" and "surgery". The search was completed in September 2013.

### Definition

The diagnostic features of HPS include presence of liver disease or portal hypertension, an elevated age-adjusted alveolar-arterial oxygen gradient (AaPO2), and evidence of intrapulmonary vasodilatation^1,2,3,14,17,19^. It is classically defined by a widened alveolar-arterial oxygen gradient (AaPO2) on room air (>15 mmHg, or >20 mmHg in patients >64 years of age) with or without hypoxemia resulting from intrapulmonary vasodilatation in the presence of hepatic dysfunction or portal hypertension ^4,17,18^. In the presence of coexisting cardiac or pulmonary disease, establishing a diagnosis of HPS can be difficult ^19^.

### Clinical manifestations

Involve respiratory findings associated with chronic liver disease. The insidious onset of dyspnea, particularly on exertion, is the most common complaint, but is not specific**. **Others symptoms may be present as platypnea and orthodeoxia^5,6^. Spider angiomata are commonly reported but are frequently seen in cirrhotic patients without HPS. Finally, clubbing and distal cyanosis, when present in the setting of liver disease or portal hypertension, should raise suspicion^2^. The majority of patients with HPS are either asymptomatic, particularly if diagnosed during evaluation for liver transplantation. Some cases develop the insidious onset of dyspnea^4,8^.

### Diagnosis

HPS diagnosis depends initially on the presence of liver disease or portal hypertension, an elevated age-adjusted alveolar-arterial oxygen gradient (AaPO_2_), and evidence of intrapulmonary vasodilatation^7^. It can normally be diagnosed with non-invasive tests. Its diagnosis is suspected based on history and physical exam; arterial blood gas analysis should be performed while breathing room air on pulse oximetry^2,4^. An elevated alveolar-arterial gradient and decrease in arterial blood gas occurs due to the dilatation of pulmonary vasculature leading to shunt with ventilation-perfusion mismatch ^17^.

The arterial blood gas reveals an elevated age-adjusted AaPO_2_ with or without hypoxemia. In detecting gas exchanges abnormalities, chest radiography and pulmonary function tests for evaluation the presence of other pulmonary abnormalities should be performed. The transthoracic microbubble contrast echocardiography is the preferred screening test for intrapulmonary vasodilatation. Pulmonary angiography, technetium-labeled macroaggregated albumin scan and chest computerized tomography could be useful in some specific situations^9,11,14,16,19^.

### Classification

The ERS Task Force has proposed a classification using arterial oxygen tension (PaO_2_) to stage the severity of HPS: PaO_2_<50 mmHg indicates very severe; PaO_2_50 <60 mmHg suggests severe; and a PaO_2 _60 and <80 mmHg corresponds with moderate stages^17^.

Krowka et al.^7^ demonstrated two angiographic patters: type I, or diffuse, normal vessels or fine diffuse spidery arterial vascular abnormalities and type II, or focal, more infrequent, consisted of similar focal arteriovenous communications (Figure 1). Patients with advanced type I or type II, may exhibit a poor response to oxygen breathing^7^.

**FIGURE 1:** Hepatopulmonary syndrome classifications

Classification ERS Task Force^17^	Arterial oxygen tension (PaO_2_)
Very severe	<50 mmHg
Severe	≥50 PaO_2_ <60 mmHg
Moderate	≥60 PaO_2_ <80 mmHg
**Krowka MJ et al. 1992^7^**	**Angiographic patters**
Type I	Diffuse, normal vessels or fine diffuse spidery vascular abnormalities
Type II	Focal, more infrequent, similar focal arteriovenous communications

### Treatment

The oxygen supplementation keeps a mainstay of therapy with a PaO_2_<60 mmHg or with oxigen desaturation exercise-induced^8,16^. The transjugular intrahepatic portosystemic shunt (TIPS) had limit utility in HPS and need more clinical trials to define the efficacy^15^. There are currently no effective medical therapies for HPS.

**Figure 2:**
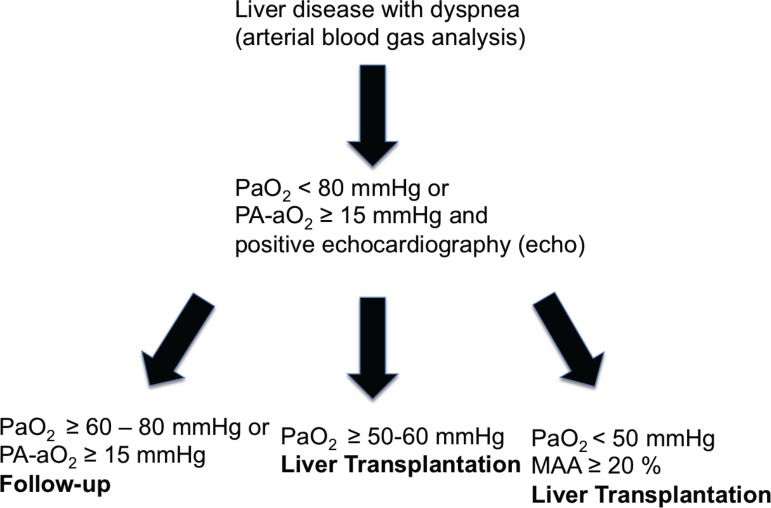
Hepatopulmonary syndrome therapeutic algorithm

Hepatopulmonary syndrome algorithm therapeutic proposed by ERS Task Force is detailed in Figure 2.

Liver transplantation is the only effective established therapy for HPS ^4,8^. The total resolution or significant improvement in gas exchange post-liver transplant is observed in more than 85% of reported patients and arterial hypoxemia normalization after transplantation is variable and may be superior to one year ^4,19^.

### Outcomes

The causes of death in patients with HPS were mainly due to complications of hepatocellular dysfunction and portal hypertension, and correlated with the severity of hypoxemia. It has been reported that HPS can increase the mortality of patients with liver cirrhosis, especially in those with severe hypoxemia^6,11,14,19^.

Mortality after liver transplantation also appears to be higher in patients with HPS compared to those without. The strongest predictors of post-liver transplantation mortality were the preoperative PaO_2_<50 mmHg alone or in combination with a macroaggregated albumin shunt fraction of 20%^3,8^. A prospective study found that those with severe HPS (PaO_2_<50 mmHg) had an important increase in post-liver transplantation mortality^3^.

HPS increases mortality and liver transplant outcomes and may worsen in cases of advanced stages. Therefore, the worldwide centers may improve the increase priority for liver transplantation in patients with HPS and significant hypoxemia. The MELD score does not apply and reduce the survival in waiting liver transplant list patients in those patients that HPS affect decreasing quality of life^3,4,6,8^. Liver transplantation has been considered as the only therapy established to reverse intrapulmonary vasodilation, but the postoperative mortality is still high in patients with a partial pressure of oxygen lower than 50 mmHg. A better understanding of the pathophysiological mechanism underlying HPS will help to better guide its treatment^1,2,17,18^.

## CONCLUSION

The severity of HPS is an important predictor to determine survival, the better moment and the risks for liver transplantation. The liver transplantation still remains the only effective therapeutic modality.
